# Fighting Obesity Through the Built Environment

**DOI:** 10.1289/ehp.112-a616

**Published:** 2004-08

**Authors:** Julie Wakefield

Although it’s easy to point the finger at everyone from Ronald McDonald to Bill Gates, no one entity or factor is specifically to blame for the nation’s raging obesity epidemic, according to speakers at the first-ever national conference on obesity and the built environment, held in late May in Washington, D.C. But every community can do something to combat it, participants agreed.

The built environment includes all aspects of the environment that are modified by humans, including homes, schools, workplaces, parks, industrial areas, and highways. Participants at Obesity and the Built Environment: Improving Public Health Through Community Design first probed how various aspects of the built environment currently contribute to obesity by affecting eating and physical activity habits and facilitating an increasingly sedentary lifestyle. Then participants discussed how the built environment can be changed to combat obesity, and how environmental health research and interventions can impact this growing public health problem.

The conference brought together researchers, planners, health care providers, developers, policy makers, and community and business leaders to develop agendas for future research and policy implementation, and to facilitate partnerships among these disciplines—goals that primary organizer Allen Dearry, NIEHS associate director of research coordination, planning, and translation, said the 600 attendees successfully realized by the meeting’s end. Highlighting evidence-based strategies for intervention, the conference also pointed to the need for interagency cooperation at all levels of government and for efforts to inform elected officials on the subject.

The conference was sponsored by the NIEHS. Its planning committee included officials from other federal agencies such as sister NIH institutes, the Department of Health and Human Services (DHHS), the Department of Housing and Urban Development (HUD), and the Centers for Disease Control and Prevention (CDC), as well as from academia.

## A Spreading Epidemic

In the United States and elsewhere, the obesity epidemic is spreading virtually unchecked. Today, about two-thirds of Americans are overweight (defined as having a body mass index of 25 or more), and one-third are obese (with a body mass index of 30 or more), said NIEHS deputy director Samuel Wilson, citing figures from the CDC’s National Health and Nutrition Examination Survey. If the trend continues, 75% of the U.S. population will be overweight within the next five years, and 40% will be obese. Meanwhile, the cost of treating obesity-related illnesses and conditions such as cardiovascular disease, cancer, and type 2 diabetes mellitus will exceed $76 billion annually in direct costs, by some estimates. When indirect costs such as lost wages are factored in, the number already exceeds $117 billion a year, according to the CDC.

In 2001, U.S. surgeon general David Satcher issued the Call to Action to Prevent and Decrease Overweight and Obesity, which has largely gone unheard. In fact, the average American adult has continued to gain 1–2 pounds a year since then, according to James O. Hill, director of the Center for Human Nutrition at the University of Colorado Health Sciences Center, and colleagues, writing in the 7 February 2003 issue of *Science*. And the obesity rate continues to climb among young people, currently affecting about 15% of children aged 6–18. The surgeon general asserted that today’s youth may be the first generation not to outlive their parents. As it is, an estimated 300,000 deaths per year may be attributable to obesity in the United States alone. Meanwhile, the World Health Organization added “overweight/obesity” to its list of the top 10 preventable health risks worldwide.

“The highest rates of obesity are found among populations with the highest poverty rates and the least education,” especially among women, said Adam Drewnowski, director for the Center for Public Health Nutrition at the University of Washington. Yet all income and education groups are steadily becoming more obese. Not only socioeconomic phenomena but also features of the built environment limit access to healthy diets, he explained to participants.

NIH director Elias Zerhouni sees the growing epidemic as an evolutionary challenge to our species. “Our intelligence and our ability to understand through science and technology and the development of industry [has enabled] us to change our environment at speeds that our natural genetic evolutionary forces are unable to adapt to,” he explained. For example, 80% of our genes are tuned to respond to food scarcity, but only 20% are designed to maintain weight in a normal range, said Zerhouni.

## Schools, Workplaces, and Communities at Large

Although obesity, like most other chronic health problems, is caused by complex interactions between genetics and environmental factors, the rapid increase in obesity over the past 30 years strongly suggests that environmental influences are responsible for this trend; the conference primarily focused on the environmental component. “Our built environment promotes a sedentary lifestyle today,” said Wilson, “and in addition to obesity there are many other attendant health challenges. . . . If we better understand the linkages between obesity and the built environment, we can create communities and workplaces that promote health and also promote well-being, an important feature in overall health.”

The conference covered obesity and the built environment in the context of three cross-cutting themes: schools and children; communities and families; and worksites, employers, and employees. In their presentations, speakers addressed a number of key questions: How do we develop, implement, and evaluate more “walkable” communities, where it is not necessary to drive everywhere due to sprawl and other poor design decisions? How do we create and assess incentives to encourage necessary changes at both the community and individual level? And how do we promote more physical activity and determine its effectiveness in maintaining a healthy weight? Across these themes, participants identified key environmental factors, from the intensive marketing of unhealthy foods, to the cultural belief that junk food tastes best, to the lack of full-service supermarkets and other nutritious food outlets in many neighborhoods, to poorly designed communities that discourage walking, biking, and other physical activity.

Part of the environmental component of obesity is an overall package of unhealthy lifestyle behaviors that contribute to the problem. These behaviors include anything that encourages a more sedentary lifestyle (such as playing video games excessively) and eating excess calories (such as a high-sugar, high-fat diet). Often the development of these behaviors starts in childhood, speakers stressed, and childhood obesity is strongly associated with adult obesity.

Many economic and political forces contribute to the problem, from budget cuts that slash school physical education and sports programs to the proliferation of vending machines on school campuses. Candy and snack food manufacturers, soft drink bottlers, and fast-food restaurants heavily market in schools, and many schools depend on revenues from these and other sources, such as annual fund-raisers selling doughnuts and candy bars. “The smartest people in the country are paid l o t s o f m o n e y t o manipulate children into behaviors that may be harmful to their health,” said Alex Molnar, director of the Education Policy Studies Laboratory at Arizona State University. And the changes have become systemic, he said, pervading the National School Lunch Program, school fundraisers, and more: “Corporate America has turned principals, teachers, and other school officials into cheerleaders that reinforce this behavior.”

Many issues regarding access to nutritious food spill over into the community at large as well. The evidence is clear that wholesome foods such as lean meats and fresh produce often cost more and that lower-cost diets are often high in starches, added sugars, and added fats, which are known to contribute to weight gain, according to Drewnowski and other conference speakers.

Emerging evidence further shows a direct association between community design and residents’ levels of physical activity. The likelihood of obesity declines with increases in mixed land use, but rises with increases in time spent in a car per day, according to recent results presented by Lawrence Frank, an associate professor of community and regional planning at the University of British Columbia. Every 30 additional minutes spent in a car was linked with a 3% increase in the risk of obesity in a recent study of nearly 11,000 Atlanta residents. “Taking into account multiple outcomes [such as residential density, land use mix, and commuting time] will likely help to explain the variation within individual outcome measures such as body mass index,” Frank explained. The study appears in the August 2004 issue of the *American Journal of Preventive Medicine*.

Obesity has become a growing concern for employers, as well, in terms of controlling health care costs and maintaining worker productivity. The numbers are daunting. Nationally, obesity costs U.S. companies more than $13 billion a year, including $8 billion for health insurance, $2.4 billion for sick leave, $1.8 billion for life insurance, and another $1 billion for disability insurance, according to the 2001 surgeon general’s Call to Action.

In addition to the increased use of health services by obese employees, “employees and employers alike incur additional costs from the impact of obesity on absenteeism, which results in lost employee income and lower corporate profits,” said David Chenoweth, president of Health Management Associates, a New Bern, North Carolina, health care research and consulting firm. In fact, obese workers are almost twice as likely to be frequently absent as people of a healthy weight, according to results by Brigham Young University health promotion professor Larry Tucker, published in the January/February 1998 *American Journal of Health Promotion*. The 2001 Call to Action noted that obesity-related illnesses cost employers 39.3 million lost workdays, 239 million days of reduced productivity, and 62.7 million doctor visits annually.

## A Growing Body of Evidence

Overall, research into the links between obesity and the environment is in its infancy. To begin with, researchers to a large extent still can’t clearly define what a healthy diet is—witness the debate over low- and high-carbohydrate diets. And the questions only get more complex from there. Relationships between community design, patterns of social interaction, and the formation of a sense of community cooperation are all factors, as are aspects of safety and security, air and water quality, mental health, and more.

“Right now, we don’t really know what a healthy environment looks like,” said Hill. As a first step to achieving this yet-undefined environment that facilitates healthy lifestyles and healthy weights, Hill invited participants to envision what it would look like. Researchers admit much work remains to figure out exactly how obesity and the built environment are connected. Moreover, there are differences between what works for adults and what works for children as far as encouraging exercise goes. Still, “while we can’t safely say that certain changes in community design will lead to increases in physical activity, we can safely say that certain changes in community design will increase *opportunities* for physical activity,” said Susan Handy, an associate professor of environmental science and policy at the University of California, Davis.

Successful strategies require governments and local communities to work together to initiate programs in schools, workplaces, and communities, and to involve food producers, industries, and consumer associations. Examples of successful partnerships with industry that target physical activity and obesity include Gatorade’s “Get Kids in Action” (which has research and education components, as well as outreach to elementary and middle school children), Nike’s “NikeGO” (which funds physical activity programs and facilities for children), and General Motors’ “Just a Bit Gets You Fit” (which emphasizes the concept of exercising in manageable chunks of time). All three work to change lifestyles and behaviors through interventions at schools or worksites.

Studies such as a review article in the November/December 2001 issue of the *American Journal of Health Promotion* reveal that these and other interventions can be effective. “Social” marketing, which uses conventional marketing and advertising approaches to promote a change in behavior in a certain population (for example, those at risk for becoming overweight or obese), can help reverse trends in weight gain. Food labeling has also been shown to decrease caloric intake and fat consumption. Moreover, reducing prices of healthier foods increases their sale.

Stronger links still need to be forged between seemingly disparate disciplines, speakers stressed—issues that seem unrelated to obesity may, in fact, be connected. In addition, developers and planners should begin measuring and accounting for the health impact of proposed land use plans and future development projects. “Smart growth plans and policies need to be more explicit about addressing health,” said Marya Morris, a senior research associate at the nonprofit American Planning Association. For example, walkability should be factored in to school siting, just as creating bike trails and adequate walkways should be an inherent part of road and highway construction. “The lack of action on these issues is due in part to the lack of understanding by planners and others about the health consequences of how we shape the built environment,” Morris said.

Although much work remains, a growing body of evidence suggests that well-designed health promotion and disease prevention programs can improve workers’ health, morale, work relations, and productivity, as well as lessen disease risk, save businesses money, and boost financial performance of organizations, reported Ron Goetzel, vice president of consulting and applied research for The Medstat Group, a market intelligence firm. DHHS secretary Tommy Thompson suggested that all employers set aside time for their employees to exercise. Thompson believes better work-place practices should start in the federal government with its health agencies. And above all, public health workers and policy makers should practice what they preach by taking the stairs more, wearing a pedometer to count daily steps, losing excess weight, and improving their diets—even taking breaks through the day to do push-ups, and more. “I want to transform the American health system,” he declared, “and small steps can make a difference.”

A consensus emerged from the conference that complex environmental health problems require an integrated multilevel response strategy. “We cannot succeed without a comprehensive, multipronged view about the problem of obesity, including the relationship not only with our genes and biology but with our environment, which is changing at a speed that overwhelms our ability to adjust to it,” Zerhouni said.

Conference participants agree that prevention is critical for children, families, communities, and workplaces because obesity has been difficult to treat thus far. NIEHS director Kenneth Olden called for greater investments in the prevention of obesity and the translation of science-based information into effective policy and action for the public. And importantly, added Olden, “Somebody has to step out and bring all these agencies together to make sure we address this important public health issue.”

## Figures and Tables

**Figure:**
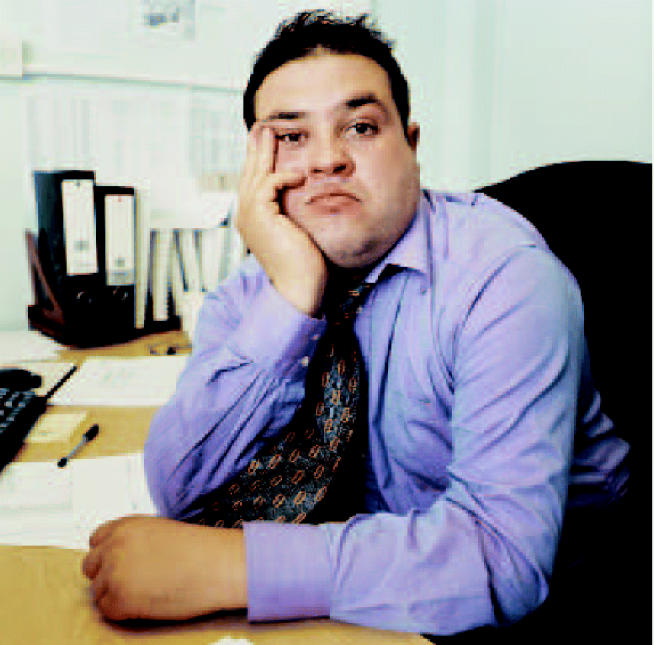
**A large work in progress.** With their junk food vending machines, restrictive schedules, and stationary tasking, workplace environments are contributing to the obesity epidemic.

**Figure:**
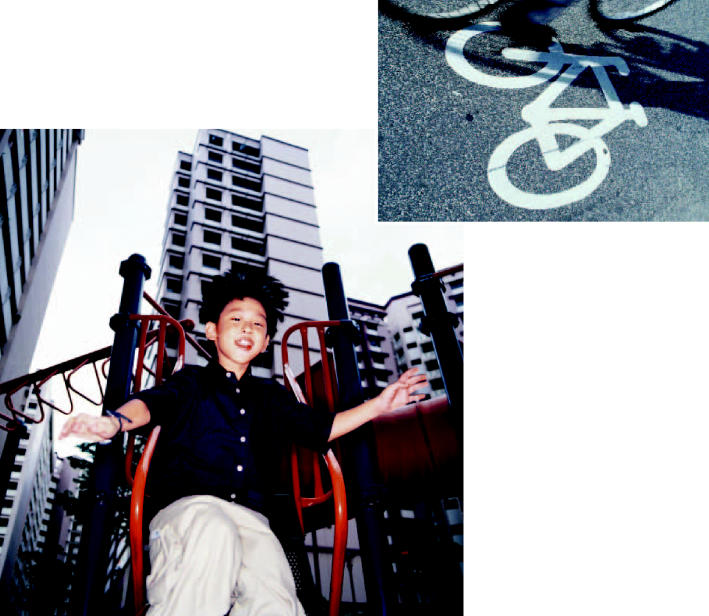
**A place for play.** Playgrounds and bike lanes have sometimes been viewed as frivolous or out of place in the urban environment, but new research shows that making it convenient to have an active lifestyle may go a long way toward preventing serious—and expensive—health problems.

